# Profiling family resilience in chronic obstructive pulmonary disease: a network analysis approach to identifying intervention targets

**DOI:** 10.3389/fpsyg.2026.1932841

**Published:** 2026-10-06

**Authors:** Qiufeng Peng, Nan Zhang, Jia Liu, Zheng Tian, Yong Liu, Yimiao Li, Xi Yu, Yuan Liu, Jingchun He, Lan Wang

**Affiliations:** 1 Department of Respiratory and Critical Care Medicine, The Fourth Central Hospital Affiliated to Tianjin Medical University, Tianjin, China; 2 Department of Ophthalmology, Peking University People’s Hospital, Beijing, China; 3 Beijing Key Laboratory of Ocular Disease and Optometry Science, Peking University People’s Hospital, Beijing, China; 4 School of Nursing, Capital Medical University, Beijing, China; 5 Department of Nursing, Tianjin Eye Hospital, Tianjin Key Lab of Ophthalmology and Visual Science, Tianjin Eye Institute, Clinical College of Ophthalmology, Tianjin Medical University, Tianjin, China; 6 State Key Laboratory of Experimental Hematology, National Clinical Research Center for Blood Diseases, Haihe Laboratory of Cell Ecosystem, Institute of Hematology & Blood Diseases Hospital, Chinese Academy of Medical Sciences & Peking Union Medical College, Tianjin, China; 7 Department of Respiratory and Critical Care, Tianjin First Central Hospital, Tianjin, China; 8 Tianjin Medical University General Hospital, Tianjin, China, Tianjin, China; 9 School of Nursing, Tianjin Medical University, Tianjin, China

**Keywords:** caregivers, COPD, family resilience, network analysis, patients

## Abstract

**Aim:**

This study aimed to explore the status of and the factors influencing family resilience among patients with chronic obstructive pulmonary disease (COPD) and caregivers and to construct a related structural model and system of influencing factors for family resilience based on network analysis methods. Furthermore, the study aimed to identify the core variables in the network, explore the interaction mechanisms and pathways of different variables, and provide an intervention basis and direction for the improvement of resilience in COPD families.

**Design:**

The study employed a cross-sectional survey design.

**Methods:**

A total of 221 COPD patient–caregiver dyads were recruited using convenience sampling from April 2023 to November 2024 at two hospitals in Tianjin, China. SPSS 26.0 was used to perform descriptive statistics, univariate analysis, correlation analysis, and multivariate analysis. R 4.2.1 was used to construct the network of family resilience dimensions, the item network, and the network models of family resilience and its influencing factors among COPD patients and caregivers.

**Results:**

Family communication and problem-solving exhibited the highest centrality in the family resilience network, showing the strongest correlation with caregivers’ ability to maintain a positive outlook. In addition, significant patient–caregiver correlations were identified in the dimensions of family communication and problem-solving. In the item network of family communication and problem-solving among COPD patients and caregivers, the node FCPS19 (We discuss problems and feel good about the solutions”) exhibited the highest centrality, while the strongest correlation was observed between FCPS19 and FCPS20(“We discuss things until we reach a resolution”). A statistically significant difference in the strength of the node FCPS8 (“We can ask for clarification if we do not understand each other”) was observed between COPD patients and caregivers. In the network of family resilience and its influencing factors among COPD patients and caregivers, family communication and problem-solving exhibited the highest centrality. The strongest correlation was observed between the family concern of caregivers and the family communication and problem-solving of patients. A statistically significant difference in the strength of family communication and problem-solving was observed between spousal caregivers and offspring caregivers.

**Conclusion:**

Interventions focused on improving family communication and problem-solving methods can enhance family resilience among patients and caregivers, especially offspring caregivers. Both patients and caregivers prefer to discuss problems with family members to find satisfactory solutions. Compared to patients, caregivers require more explanations from family members when they do not understand something. Nurses should encourage other family members to care more for caregivers, monitor changes in family communication and problem-solving between patients and caregivers, and explore their potential connections.

## 1 Introduction

Chronic obstructive pulmonary disease (COPD) is a heterogeneous pulmonary disorder typically characterized by progressive airflow limitation (Celli et al., 2022). Globally, it ranks as the third leading cause of death, following ischemic heart disease and stroke (WHO, 2019). The continuous airflow limitation of COPD and the complications in the development of the disease (Fernández-García et al., 2020), such as pulmonary hypertension (Zhang L. et al., 2022; Zhang W. et al., 2022), cardiovascular and cerebrovascular diseases (Gulea et al., 2022), and systemic musculoskeletal diseases (Chen et al., 2019), have a serious impact on the physical and mental health and self-care ability of patients and determine the long-term and difficult prevention and treatment process. With disease progression, COPD patients gradually lose their ability to live independently (Fernández-García et al., 2020), and disease management has become a part of caregivers’ daily life. During the home-based management of long-term chronic diseases, patients and caregivers are confronted not only with substantial physical burdens and economic pressure but also with multiple severe psychosocial challenges. Patients are prone to negative psychological outcomes, such as anxiety and depression, resulting from loss of independence, social isolation, and feelings of helplessness (Gamze et al., 2016). Meanwhile, long-term, high-intensity, and uninterrupted caregiving can disrupt families’ original lifestyles and interpersonal interaction patterns, rendering caregivers vulnerable to social isolation and triggering a range of social adaptation-related difficulties, including family conflicts and strained intimate relationships (Gholami et al., 2025).

Family constitutes an important source of social support for patients with COPD (Johansson et al., 2025). Family-provided care enhances treatment adherence, improves exercise tolerance, and reduces healthcare utilization among COPD patients (Nakken et al., 2015). However, the unpredictable acute exacerbations and recurrent episodes of COPD fundamentally disrupt family life (Aasbø et al., 2016), leaving patients and caregivers barely able to detach themselves from the demands of managing the disease. Caregivers often become fully immersed in their caregiving roles, as the need to provide round-the-clock care for patients leaves them with little to no respite. The multifaceted pressures stemming from the disease continually test the family’s coping capacity and trigger negative changes or deterioration in intimate relationships (Simpson et al., 2010). Strong family functioning plays an important role in enhancing family members’ ability to cope with chronic diseases and can also have a positive impact on patients’ disease management and psychological adjustment (Liu, 2018).

Family resilience, which considers the family as a whole and refers to the ability to cope with life changes or multiple stressors, is a powerful determinant of family functioning in patients with chronic diseases (Zhang L. et al., 2022; Zhang W. et al., 2022) and plays an important role in facilitating family members’ adaptation to the disease. Family resilience depends on many factors related to family life and encompasses not only the ability to survive crisis but also the potential to grow in the face of adversity (Black and Lobo, 2008; Höltge et al., 2021). Maintaining a balance between the needs of family members and constraints allows the family to adapt to the impact of the disease on their lives (Kim and Ahn, 2022) and ensures that the family is a place conducive to recovery from the disease, thereby promoting patients’ physical well-being and reducing the burden on the caregiver (Bagnasco et al., 2021). An in-depth understanding of the interaction mechanisms and pathways through which family resilience interacts with its associated variables may facilitate the development of targeted interventions to enhance disease coping strategies.

Currently, most studies on COPD focus primarily on individuals, examining the physical and mental changes experienced by patients or caregivers. However, dealing with major diseases is not simply a process of using individual advantages; rather, it involves a complex interplay of dual effects (Kuang et al., 2023). Network analysis is an emerging method in psychology, in which a network is composed of two key elements: “nodes,” which represent variables or symptoms, and “edges,” which represent the interactions between variables or symptoms (Boccaletti et al., 2006). Network analysis is driven by data and does not depend on prior assumptions. The interaction of all relevant variables can be formed into a complex network of mutual influence (Galderisi et al., 2018), reflecting the mechanisms and pathways of interaction among variables. Family resilience depends on many factors related to family life, but existing studies have only unilaterally explored the relevant variables associated with family resilience of patients or caregivers. The complexity and interrelationship of the problems experienced by patients and caregivers throughout the progression of chronic diseases remain insufficiently understood. The visualization of network analysis can be closer to the complex reality faced by patients and caregivers in the process of home care for chronic diseases. In addition to the correlation and effect of variables, network analysis can also identify the most central or important variable and influence other variables in the family resilience network through the activation of the central node, making the intervention more targeted and effective. In addition, more variables are prone to false correlations, and the partial correlation network used in network analysis applies a regularization process to reduce the occurrence of false-positive correlations (Epskamp and Fried, 2018). Therefore, the network analysis method can explore the interaction mechanisms and pathways of chronic disease family resilience and related factors from a new perspective.

This study employed a dual-perspective approach to examine the current state of family resilience among patients with COPD. Utilizing network analysis, we constructed an integrated framework to map: (1) interactions among dimensions of family resilience, (2) item-level association networks, and (3) the interplay between family resilience and its psychosocial determinants in both patients and caregivers. Through this approach, we identified core variables, elucidated their dynamic interrelationships, and established evidence-based targets for family-centered COPD management interventions.

## 2 Methods

### 2.1 Research design

This study employed a quantitative, cross-sectional research design.

### 2.2 Participants and settings

A total of 221 patients with COPD and their caregivers were recruited from two hospitals in Tianjin, China. Both the patient and caregiver in each dyad were required to meet the inclusion and exclusion criteria simultaneously to be included as research participants.

The inclusion criteria for patients were as follows: (1) meeting the diagnostic criteria specified in the *Guidelines for the Diagnosis and Treatment of Chronic Obstructive Pulmonary Disease (Revised Edition 2023)* (Global Initiative for Chronic Obstructive Lung Disease, 2023), (2) being 18 years of age or older, (3) being willing to participate in the study, and (4) having effective communication skills. The exclusion criteria for patients were as follows: (1) severe physical or mental illness and (2) being in poor health and unable to cooperate with the investigation.

The inclusion criteria for caregivers were as follows: (1) being 18 years of age or older, (2) being a primary caregiver and residing with the patient, (3) providing core care for a minimum of 5–40 h per week for at least 5 days (Liu, 2018), (4) possessing effective communication skills, and (5) being willing to participate in the study. The exclusion criteria for caregivers were as follows: (1) severe physical or mental illness and (2) receiving compensation for caregiving.

### 2.3 Sample size calculation

The sample size was estimated using the formula *N* = 4Uα^2^S^2^ /δ^2^ (Ni et al., 2010), with an allowable error δ set at 0.25S and 0.50S, α = 0.05, and Uα = 1.96. According to our pilot experiment, the mean family resilience score of patients was 91.64, and the standard deviation was 6.12. The corresponding allowable errors were 1.53 and 3.06. Substituting these values into the formula yielded a raw sample size ranging from 62 to 246. After accounting for a 20% rate of invalid questionnaires, the required sample size for patients was 75 to 296. The mean family resilience score among caregivers was 96.61, with a standard deviation of 9.22. The corresponding allowable errors δ were 2.31 and 4.61. Substituting these values into the formula yielded a raw sample size ranging from 63 to 246. After accounting for a 20% rate of invalid questionnaires, the required sample size for caregivers was 76 to 296.

### 2.4 Instruments

#### 2.4.1 General information questionnaire

This questionnaire was self-developed on the basis of previous relevant studies and literature references, combined with deliberations within the research team (Yi et al., 2021). The general information collected from patients included demographic characteristics (gender, age, education level, marital status, occupation, medical payment method, number of offspring, family atmosphere, and per capita monthly household income) and clinical characteristics (COPD severity, disease duration, number of complications, smoking status, and number of exacerbations in the past year). For caregivers, the collected information included caregiving-related factors (relationship with the patient, chronic disease status, daily length of caregiving, duration of caregiving, main household decision-maker, and family breadwinner) and the same demographic characteristics collected from patients.

#### 2.4.2 Shortened Chinese version of the family resilience assessment scale

Family resilience was assessed using the Family Resilience Assessment Scale (FRAS) (Li et al., 2016). The 32-item Chinese version includes three dimensions: Family communication and problem-solving (23 items), utilizing social resources (3 items), and maintaining a positive outlook (6 items). Participants rated each item on a 4-point Likert scale ranging from 1 (strongly disagree) to 4 (strongly agree). The higher the score, the higher the overall level of family resilience. The Cronbach’s α coefficients for patients and caregivers were 0.948 and 0.959, respectively.

#### 2.4.3 Connor–Davidson resilience scale

Resilience was assessed using the Connor–Davidson Resilience Scale (CD-RISC) (Yu et al., 2007). The 25-item Chinese version includes three dimensions: Tenacity (13 items), strength (8 items), and optimism (4 items). Participants rated each item on a 5-point Likert scale ranging from 0 (not at all applicable) to 4 (extremely applicable). The higher the score, the higher the overall level of individual resilience. The Cronbach’s α coefficients for patients and caregivers were 0.943 and 0.941, respectively.

#### 2.4.4 Zarit caregiver burden interview

Caregiver burden was assessed using the Zarit Caregiver Burden Interview (ZCBI) (Wang et al., 2006). The 22-item Chinese version includes two dimensions: Personal strain (12 items) and role strain (6 items). Participants rated each item on a 5-point Likert scale ranging from 0 (never) to 4 (always). The higher the score, the higher the level of caregiver burden. The Cronbach’s α coefficient was 0.933.

#### 2.4.5 Simplified coping style questionnaire

Simplified coping style was assessed using the Simplified Coping Style Questionnaire (Xie, 1998). The 20-item Chinese version includes two dimensions: Positive coping style (12 items) and negative coping style (8 items). Participants rated each item on a 4-point Likert scale ranging from 0 (never) to 3 (always). The higher the positive coping style, the higher the level of coping capacity. The higher the negative coping style, the lower the level of coping capacity. The Cronbach’s α coefficients for positive coping style and negative coping style dimensions were 0.881 and 0.795, respectively.

#### 2.4.6 Perceived social support scale

Perceived social support was assessed using the Perceived Social Support Scale (PSSS) (Jiang, 2001). The 12-item Chinese version includes three dimensions: Family support (4 items), friend support (4 items), and other support (4 items). Participants rated each item on a 7-point Likert scale ranging from 1 to 7. The higher the score, the higher the level of perceived social support. The Cronbach’s α coefficients for patients and caregivers were 0.939 and 0.930, respectively.

#### 2.4.7 Family concern index questionnaire

Family concern was assessed using the Family Concern Index Questionnaire (APGAR) (Lv and Gu, 1995). The Chinese version includes five items. Participants rated each item on a 3-point Likert scale ranging from 0 to 2. The higher the score, the higher the level of family concern. The Cronbach’s α coefficients for patients and caregivers were 0.901 and 0.907, respectively.

#### 2.4.8 Medical coping modes questionnaire

Medical coping was assessed using the Medical Coping Modes Questionnaire (MCMQ) (Shen and Jiang, 2000). The 20-item Chinese version includes three dimensions: Confrontation (8 items), avoidance (7 items), and acceptance-resignation (5 items). Participants rated each item on a 4-point Likert scale ranging from 1 to 4. The higher the confrontation, the higher the level of coping capacity. The higher the avoidance and acceptance-resignation, the lower the level of coping capacity. The Cronbach’s α coefficients for confrontation, avoidance, and acceptance-resignation dimensions were 0.887, 0.810, and 0.909, respectively.

#### 2.4.9 6-item self-efficacy for managing chronic disease scale

Self-efficacy for chronic disease management was assessed using the 6-item Self-efficacy for Managing Chronic Disease Scale (SES-6) (Wang et al., 2017). Participants rated each item on a scale ranging from 1 to 10. The higher the score, the higher the self-efficacy. This scale has been verified to have good applicability among Chinese populations. The Cronbach’s α coefficient was 0.943.

#### 2.4.10 General self-efficacy scale

General self-efficacy was assessed using the General Self-efficacy Scale (GSES) (Hu et al., 2014). Participants rated each item on a 4-point Likert scale ranging from 1 to 4. The higher the score, the higher the self-efficacy. This scale has been verified to have good applicability among Chinese populations. The Cronbach’s α coefficient was 0.858.

### 2.5 Data collection

This study obtained ethical approval (see Section 2.7 Ethical approval) and received support from the partner hospitals. The investigators were professionally trained and used a unified instruction language to guide patients and caregivers in completing the questionnaires. For participants who could not complete the questionnaire independently, the researchers used standard instructions to ask about the content and recorded the answers objectively and accurately. The questionnaires were checked and entered by two research assistants to control for errors. A total of 234 questionnaires were collected from April 2023 to May 2025. After excluding 13 invalid questionnaires, 221 valid questionnaires were included in the final analysis. The response rate was 94.4%.

### 2.6 Data analysis

IBM SPSS 26.0 and R4.2.1 were used for data sorting and statistical analysis. A two-tailed test was adopted, and a *p*-value of <0.05 was considered statistically significant.

#### 2.6.1 Descriptive statistical analysis

Descriptive statistics and variance analysis were performed using the IBM SPSS26.0 software. Frequencies and percentages were used to describe the enumeration data of patients and caregivers. Measurement data that followed a normal distribution were expressed as mean ± standard deviation.

#### 2.6.2 Single-factor analysis

Independent samples *t*-tests and ANOVA were performed on normally distributed data to examine differences in family resilience among patients and among caregivers within subgroups defined by general information (including demographic characteristics, clinical profiles, caregiving-related factors, etc.).

#### 2.6.3 Correlation analysis

Pearson correlation analysis was used to analyze the correlation between COPD patients’ family resilience and resilience, perceived social support, disease management self-efficacy, family concern, and medical coping style, as well as between patients’ family resilience and caregivers’ caregiver burden, resilience, perceived social support, general self-efficacy, family concern, and coping style. Pearson correlation analysis was used to analyze the correlation between caregivers’ family resilience and COPD patients’ resilience, perceived social support, disease management self-efficacy, family concern, and medical coping style. It was also used to examine the correlation between caregivers’ family resilience and caregivers’ caregiver burden, resilience, perceived social support, general self-efficacy, family concern, and coping style.

#### 2.6.4 Multiple-factor analysis

Meaningful variables from univariate and correlation analyses of family resilience among COPD patients and caregivers were included in the stepwise multiple regression analysis.

#### 2.6.5 Network analysis

The dimension and item association networks of family resilience among COPD patients and caregivers were constructed using network analysis, and the patient and caregiver networks were compared. Network models of family resilience and its influencing factors were constructed for COPD patients and their caregivers. The networks of spousal caregivers and offspring caregivers were then compared. The constructed networks were visualized, the centrality index was calculated, and the stability and accuracy of the networks were tested.

(1) A Gaussian graphical model (GGM) was used to construct a partial correlation network for the interaction of family resilience and related factors between patients and caregivers. The network was estimated using the graphical least absolute shrinkage and selection operator (GLASSO) algorithm to remove false-positive correlations (Epskamp and Fried, 2018). The extended Bayesian information criterion (EBIC) was used to shrink the edges in the network, with the tuning parameter λ set to 0.5 to preserve a more realistic node connection relationship.

(2) The qgraph package was used to visualize the network (Epskamp et al., 2012). The network was composed of nodes and edges between the nodes; the thicker the line, the stronger the connection between the two nodes. The blue edge between the nodes indicated a positive correlation, while the red edge indicated a negative correlation. Applying the Fruchterman–Reingold algorithm to network visualization, strongly correlated nodes or connections were distributed more centrally in the network, while weakly correlated nodes or connections were distributed to the periphery (Malhotra and Kempegowda, 2023).

(3) The Bootnet package was used to calculate the centrality index of the network and comprehensively compare the importance of each node within the network, including strength centrality. Strength centrality refers to the sum of the absolute weights connected to the edges of the symptom, is a measure of the overall degree of connection between a symptom and other symptoms in the network, and reflects the ability of the symptom to affect other symptoms (Epskamp et al., 2018).

(4) The stability and accuracy of the network were tested using the Bootnet package. First, 95% confidence intervals (CIs) were calculated using a non-parametric self-help method to check the accuracy of the edge weights (Epskamp et al., 2018). Relatively narrow CIs indicated that the accuracy of the edge weight estimation was acceptable. Second, the Centrality Stability Coefficient (CS-C) was calculated using a bootstrap procedure to evaluate the network’s stability. A CS-C value should not be lower than 0.25; it should preferably be higher than 0.5. Finally, differences in nodes and edges were evaluated using the bootstrap test.

(5) The Network Comparison Test package was used to evaluate the differences between networks (van Borkulo et al., 2023). First, global network strength was evaluated by comparing the sum of all edge weights between the networks. Second, the global structure of the networks was assessed by comparing the distribution of edge weights within each network. Finally, we compared the intensity difference of each edge between the networks, and the *P* values were corrected for multiple comparisons using the Holm‑Bonferroni method (*P* < 0.05).

### 2.7 Ethical approval

Ethical approval was obtained from the Ethics Committee of Tianjin Medical University (grant number TMUhME20230018). The study was conducted in compliance with the Declaration of Helsinki. This study was registered with the China Clinical Trial Registry (registration no. ChiCTR2300078559).

## 3 Results

### 3.1 Demographic characteristics

A total of 221 COPD patient–caregiver dyads were enrolled in this study. Overall, the patients were predominantly older adults, with a high proportion of male individuals, while the caregivers were mostly middle-aged adults, and the majority were spouses or immediate family members of the patients. Detailed demographic characteristics of the COPD patients and caregivers are provided in Supplementary Tables S3, S4.

### 3.2 Associations between family resilience in COPD patients and caregivers

#### 3.2.1 Association network of family resilience dimensions in COPD patients and caregivers

The network models of family resilience dimensions for patients and caregivers are shown in Figure 1, and the network edge weights are reported in Supplementary Table S1. The numbers on the lines of the network model represent the edge weights. The thicker the line of nodes in the network, the larger the edge weight coefficient, indicating that the correlation between the two nodes was stronger. The results showed that the strongest association among caregivers was between family communication and problem-solving and maintaining a positive outlook (weight = 0.53), followed by the correlation for patients (weight = 0.47). Family communication and problem-solving showed the strongest association between patients and caregivers (weight = 0.36).

**Figure 1:**
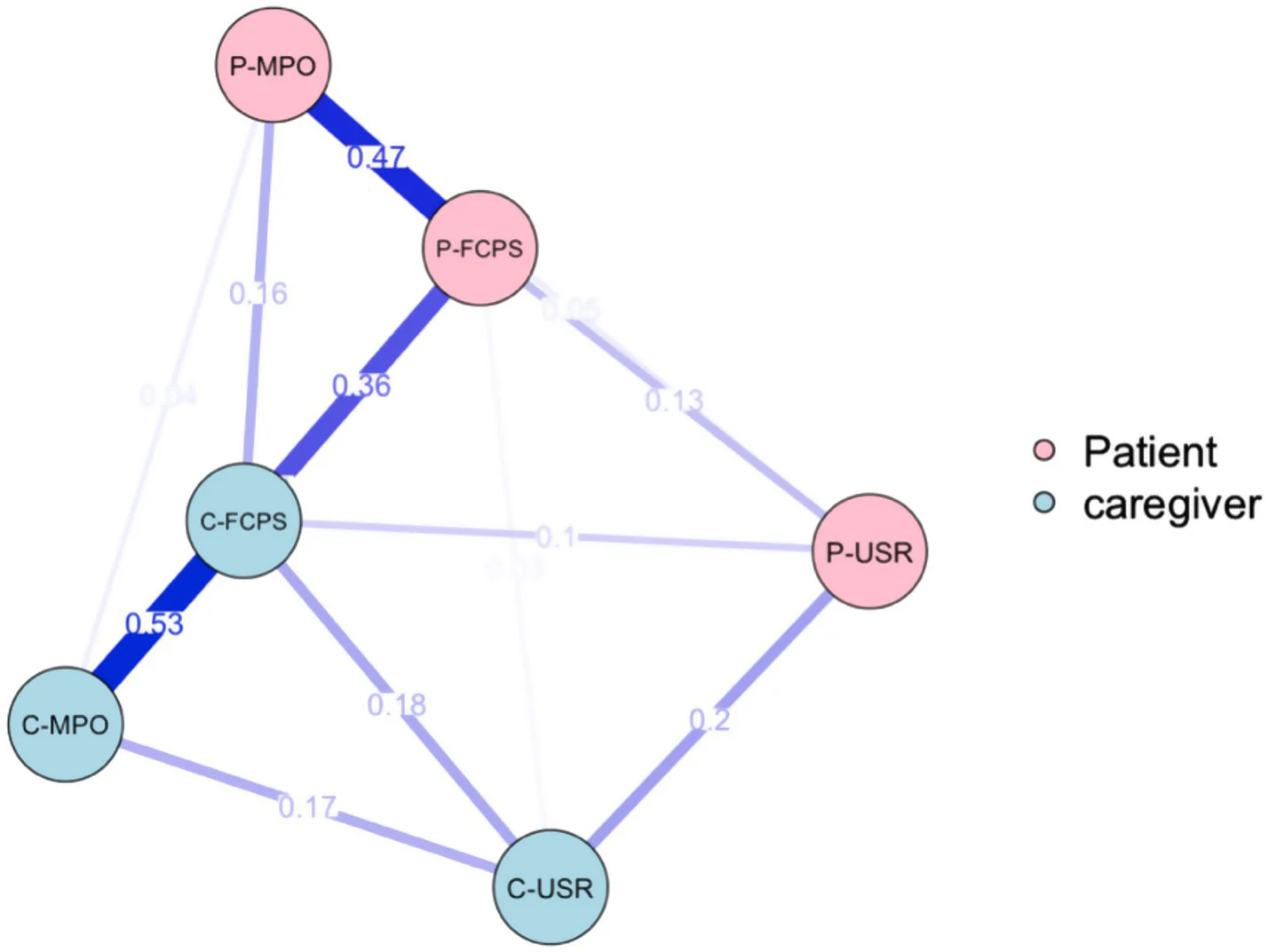
Network model of associations among family resilience dimensions (*n* = 221). P-FCPS, family communication and problem-solving among patients; P-USR, utilizing social resources among patients; P-MPO, maintaining a positive outlook among patients; C-FCPS, family communication and problem-solving among caregivers; C-USR, utilizing social resources among caregivers; C-MPO, maintaining a positive outlook among caregivers.

The centrality index of the network nodes is shown in Supplementary Figure S1. Family communication and problem-solving among caregivers showed the highest centrality (r_S_ = 1.33), followed by family communication and problem-solving among patients (r_S_ = 1.10). Network accuracy and stability are shown in Supplementary Figures S2–S5.

#### 3.2.2 Construction and comparison of item networks for family communication and problem-solving between COPD patients and caregivers

Family communication and problem-solving among COPD patients and caregivers was the most important dimension of family resilience. To further explore the correlation and importance of different items within family resilience, network association structures of family communication and problem-solving items were constructed for COPD patients and caregivers, and the networks of the two groups were compared.

The item network models of family communication and problem-solving among COPD patients and caregivers are shown in Figure 2. The network edge weights for patients are presented in Supplementary Table S2, while those for caregivers are presented in Supplementary Table S3. In the networks, node FCPS19 (We discuss problems and feel good about the solutions) and node FCPS20 (We discuss things until we reach a resolution) showed the greatest correlation (Patient weight = 0.53; Caregiver weight = 0.37). The Network Comparison Test showed no significant difference between COPD patients and caregivers (*M* = 0.22, *p* = 0.390). Similarly, there was no significant difference in global strength between patients and caregivers (Patients = 10.43 vs. Caregivers = 10.72; *S* = 0.29, *p* = 0.183).

**Figure 2:**
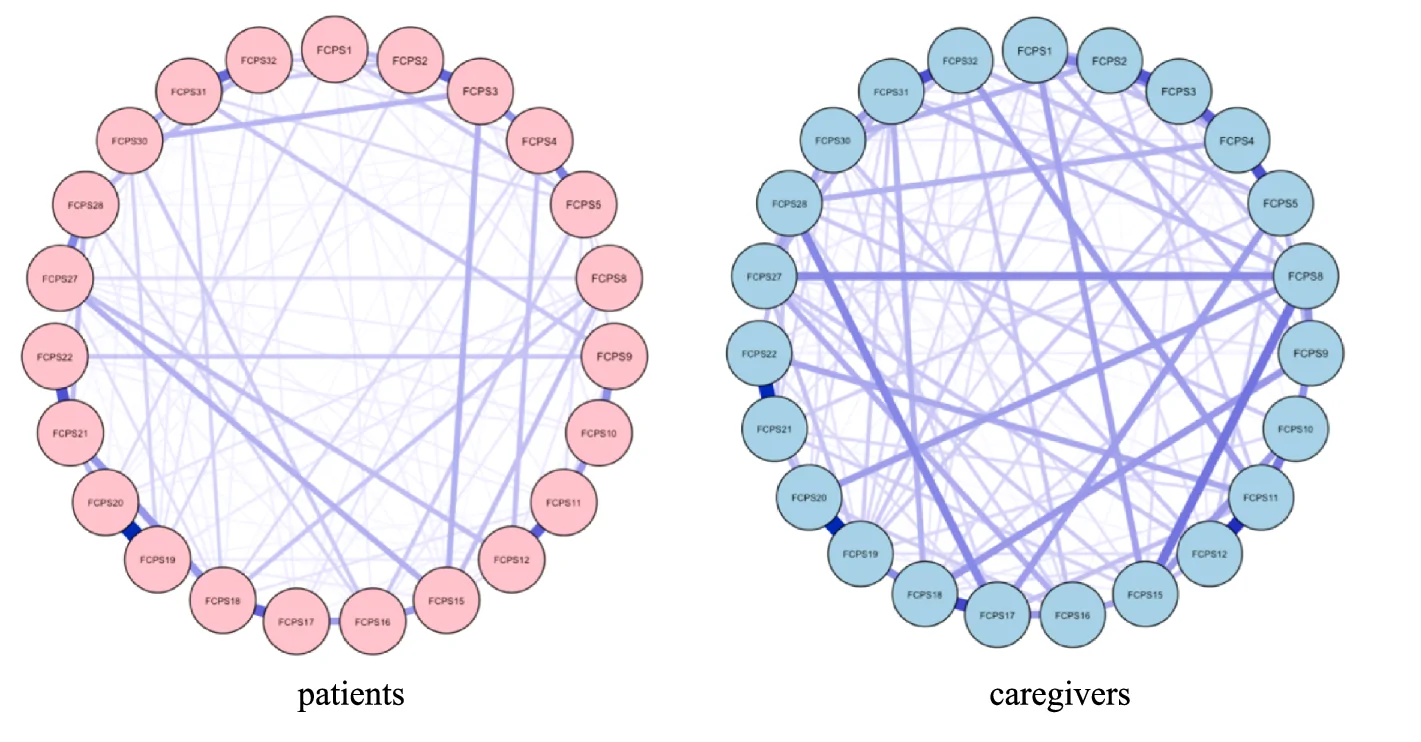
Network model of family resilience item associations (*n* = 221). FCPS1 = Our family structure is flexible to deal with the unexpected; FCPS2 = We are able to work through pain and come to an understanding; FCPS 3 = We are adaptable to demands placed on us as a family; FCPS4 = We are open to new ways of doing things in our family; FCPS5 = We are understood by other family members; FCPS8 = We can ask for clarification if we do not understand each other; FCPS9 = We can be honest and direct with each other in our family; FCPS10 = We can blow off steam at home without upsetting someone; FCPS11 = We can compromise when problems come up; FCPS12 = We can deal with family differences in accepting a loss; FCPS15 = We can talk about the way we communicate in our family; FCPS16 = We can work through difficulties as a family; FCPS17 = We consult with each other about decisions; FCPS18 = We define problems positively to solve them; FCPS19 = We discuss problems and feel good about the solutions; FCPS20 = We discuss things until we reach a resolution; FCPS21 = We feel free to express our opinions; FCPS22 = We feel good giving time and energy to our family; FCPS27 = We learn from each other’s mistakes; FCPS28 = We share responsibility in the family; FCPS30 = We try new ways of working with problems; FCPS31 = We understand communication from other family members; FCPS32 = We work to make sure family members are not emotionally or physically hurt.

The centrality index of the network nodes is shown in Supplementary Figure S6. Node FCPS19 had the highest centrality (Patient r_S_ = 1.12, Caregiver r_S_ = 1.19). The centrality of node FCPS8 (We can ask for clarification if we do not understand each other) was significantly higher in the COPD patient network than in the caregiver network (*p* = 0.002). Network accuracy and stability are shown in Supplementary Figures S7–S10.

### 3.3 Analysis of factors influencing family resilience among COPD patients and caregivers

#### 3.3.1 Differences in family resilience among COPD patients and caregivers according to general characteristics

Supplementary Table S4 shows the comparison of family resilience among COPD patients according to patients’ and caregivers’ general characteristics. Significant differences in the family resilience scores of COPD patients were observed according to patients’ age, education level, marital status, occupation, COPD severity, disease duration, number of complications, smoking status, number of acute exacerbations in the past year, medical expense payment method, number of offspring, family atmosphere, and per capita monthly household income. Significant differences in the family resilience scores of COPD patients were observed according to caregivers’ education level, marital status, occupation, relationship with patients, whether they had chronic diseases, daily length of caregiving, duration of caregiving, whether they were the primary household decision-maker, and whether they were the primary breadwinner.

Supplementary Table S5 shows the comparison of family resilience among caregivers according to patients’ and caregivers’ general characteristics. Significant differences in the family resilience scores of COPD caregivers were observed according to patients’ education level, marital status, occupation, COPD severity, disease duration, number of complications, smoking status, number of acute exacerbations in the past year, medical expense payment method, number of offspring, family atmosphere, and per capita monthly household income. Significant differences in the family resilience scores of COPD caregivers were observed according to caregivers’ gender, education level, marital status, occupation, relationship with patients, whether they had chronic diseases, daily length of caregiving, duration of caregiving, whether they were the primary household decision-maker, and whether they were the primary breadwinner.

#### 3.3.2 Correlation analysis of factors related to family resilience among COPD patients and caregivers

Supplementary Table S6 shows the results of the correlation analysis. Patients’ family resilience was positively correlated with resilience, perceived social support, self-efficacy for managing chronic disease, family concern, and confrontation (*p* < 0.001) and negatively correlated with avoidance and acceptance-resignation of patients (*p* < 0.001). It was positively correlated with resilience, perceived social support, general self-efficacy, family concern, and positive coping (*p* < 0.001) and negatively correlated with caregiver burden and negative coping (*p* < 0.001). Caregivers’ family resilience was positively correlated with resilience, perceived social support, self-efficacy for managing chronic disease, family concern, and confrontation (*p* < 0.001) and negatively correlated with avoidance and acceptance-resignation of patients (*p* < 0.001). It was positively correlated with resilience, perceived social support, general self-efficacy, family concern, and positive coping (*p* < 0.001) and negatively correlated with caregiver burden and negative coping (*p* < 0.001).

#### 3.3.3 Multivariate analysis of family resilience among COPD patients and caregivers

Table 1 shows that per capita monthly household income, number of offspring, patients’ resilience, patients’ perceived social support, and caregivers’ family concern were included in the regression equation. *R^2^* = 0.746 and adjusted *R^2^* = 0.740, suggesting that 74.0% of the total variation in family resilience of COPD patients was jointly explained by the model. Tolerance and the variance inflation factor (VIF) were used to assess multicollinearity among the variables. The results showed that tolerance values ranged from 0.340 to 0.992, while VIF values ranged from 1.008 to 2.547, both of which were below 10, indicating that there was no multicollinearity among the independent variables. The Durbin–Watson value was 1.904, close to 2, indicating that there was no autocorrelation between the residual terms.

**Table 1:** Stepwise regression analysis of family resilience among COPD patients.

Variables	*B*	*β*	*t*	*p*	Collinearity statistics	
					Tolerance	VIF
Constant value	46.853		17.386	<0.001		
Patient						
Per capita monthly household income	2.403	0.197	4.006	<0.001	0.489	2.045
Number of offspring	1.332	0.097	2.801	0.006	0.992	1.008
Resilience	0.273	0.343	5.804	<0.001	0.340	2.945
Perceived social support	0.217	0.206	3.868	<0.001	0.417	2.398
Caregiver						
Family concern	1.031	0.252	4.599	<0.001	0.393	2.547

F = 126.033, *p* < 0.001, R^2^ = 0.746, adjusted R^2^ = 0.740.

Table 2 shows that education level, avoidance and acceptance-resignation of patients, caregiver being the patient’s offspring, and resilience and family concern of caregivers were included in the regression equation: *R^2^* = 0.762 and adjusted *R^2^* = 0.756, suggesting that 75.6% of the total variation in family resilience of caregivers was jointly explained by the model. Tolerance and the VIF were used to assess multicollinearity among the variables. The results showed that tolerance values ranged from 0.353 to 0.889, while VIF values ranged from 1.125 to 2.834, both of which were below 10, indicating that there was no multicollinearity among the independent variables. The Durbin–Watson value was 1.682, close to 2, indicating that there was no autocorrelation between the residual terms.

**Table 2:** Stepwise regression analysis of family resilience among caregivers.

Variables	*B*	*β*	*t*	*P*	Collinearity statistics	
					Tolerance	VIF
Constant value	72.803		17.263	<0.001		
Patient						
Education level	2.162	0.158	4.074	<0.001	0.738	1.355
Avoidance	−0.323	−0.120	−2.469	0.014	0.472	2.119
Acceptance-resignation	−0.437	−0.151	−2.697	0.008	0.353	2.834
Caregiver						
Relationship with patients						
Offspring	2.183	0.096	2.721	0.007	0.889	1.125
Resilience	0.257	0.286	5.586	<0.001	0.424	2.361
Family concern	1.566	0.344	6.294	<0.001	0.373	2.683

F = 114.385, *p* < 0.001, R^2^ = 0.762, adjusted R^2^ = 0.756.

### 3.4 Network analysis of family resilience and its influencing factors

#### 3.4.1 Network analysis of family resilience and its influencing factors among COPD patients

To further explore the relationship between family resilience and its influencing factors among COPD patients and to identify intervention targets, we constructed a network model incorporating the statistically significant variables identified in the stepwise regression analysis of patients’ family resilience, together with all dimensions of family resilience.

Figure 3 shows the network model of family resilience and its influencing factors among COPD patients, while Supplementary Table S7 shows the network edge weights. The results showed that family concern of caregivers had the greatest association with patients’ family communication and problem-solving (weight = 0.23). The centrality index of the network nodes is shown in Supplementary Figure S11. The centrality of family communication and problem-solving was the highest among the dimensions of family resilience (r_S_ = 1.46). Network accuracy and stability are shown in Supplementary Figures S12–S15.

**Figure 3:**
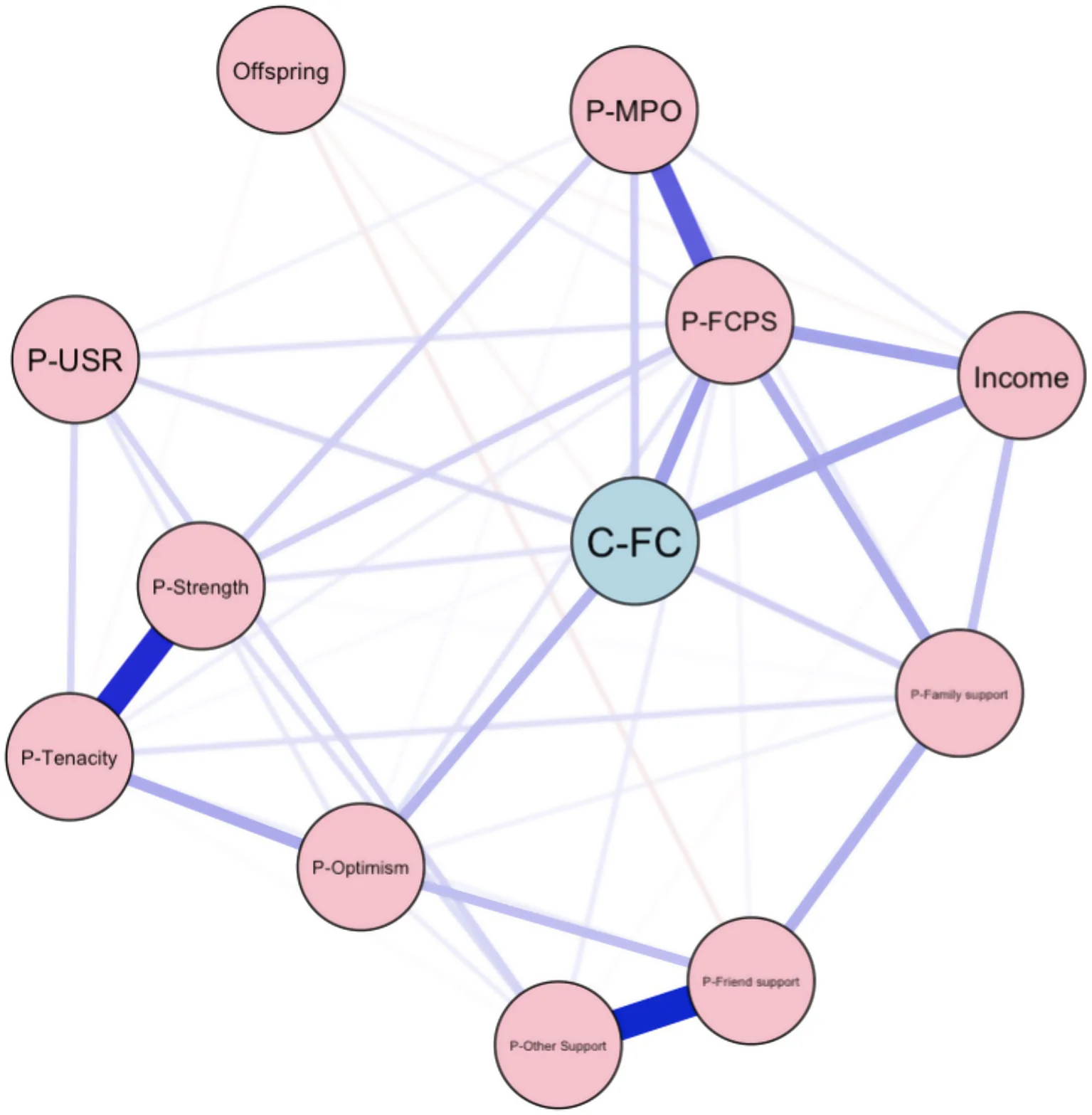
Network model of family resilience and its influencing factors among COPD patients (*n* = 221). P-FCPS = family communication and problem-solving of patients; P-USR = utilizing social resources of patients; P-MPO = maintaining a positive outlook of patients; Income = per capita monthly household income; Offspring = number of offspring; P-Strength = strength of patients; P-Tenacity = tenacity of patients; P-Optimism = optimism of patients; P-family support = family support of patients; P-friend support = friend support of patients; P-other support = other support of patients; C-FC = family concern of caregivers.

#### 3.4.2 Network analysis of family resilience and its influencing factors among caregivers

To further explore the relationship between family resilience and its influencing factors among caregivers and to identify intervention targets, we constructed a network model incorporating the statistically significant variables identified in the stepwise regression analysis of caregivers’ family resilience, together with all dimensions of family resilience.

Figure 4 shows the network model of family resilience and its influencing factors among spousal caregivers and offspring caregivers. Supplementary Tables S8, S9 show the network edge weights. In the spousal caregiver network, caregivers’ family concern had the strongest association with family communication and problem-solving among caregivers (weight = 0.50). In the offspring caregiver network, caregivers’ family concern had the strongest association with family communication and problem-solving among caregivers (weight = 0.35). The network comparison results showed that there were no significant differences in the global structure (*M* = 0.26, *p* = 0.319) and global network strength (Spouse = 4.19 vs. Offspring = 4.45; *S* = 0.27, *p* = 0.337) of family resilience between spousal caregivers and offspring caregivers.

**Figure 4:**
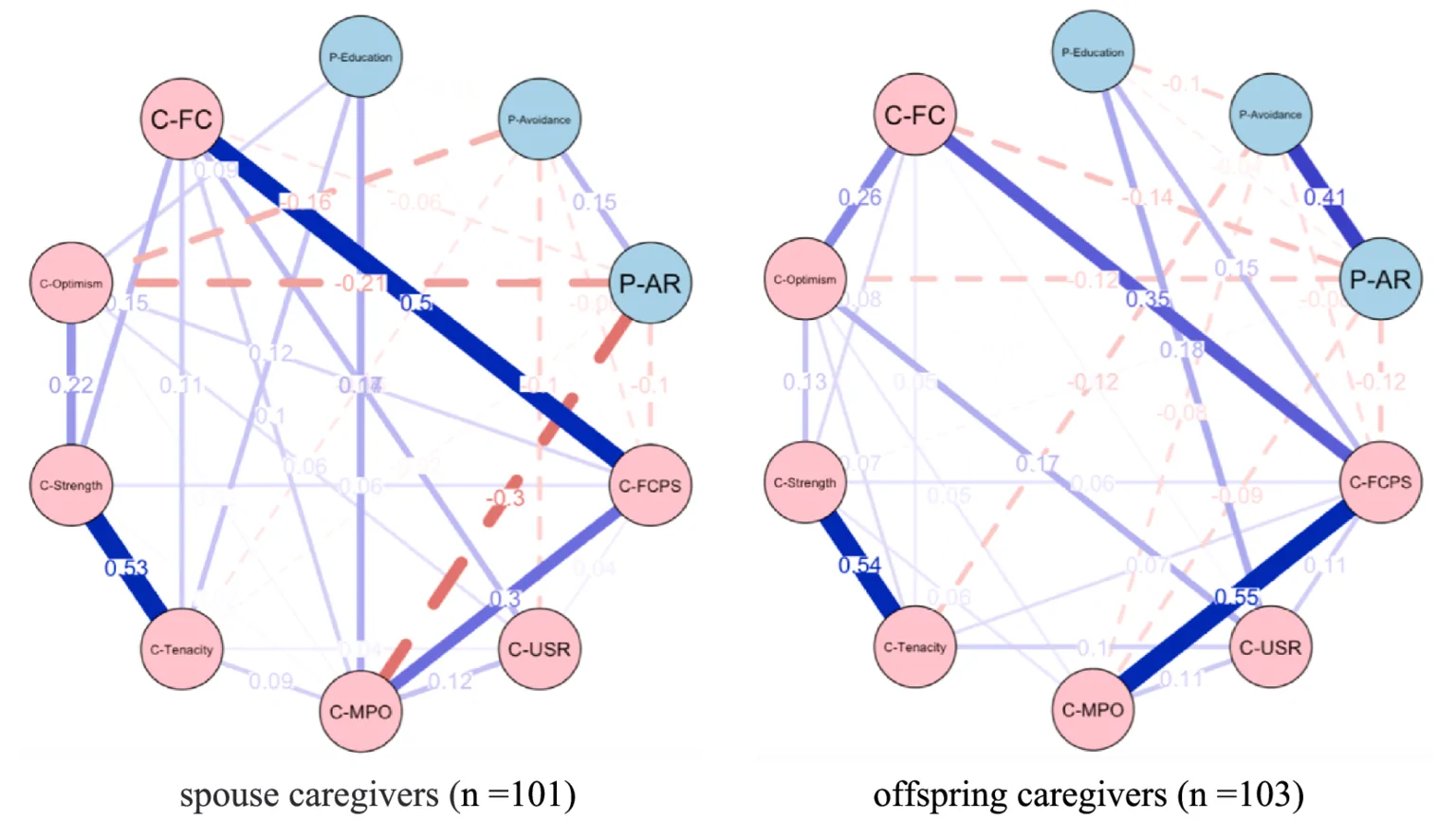
Construction and comparison of network models of family resilience and its influencing factors among caregivers. C-FCPS = family communication and problem-solving of caregivers; C-USR = utilizing social resources of caregivers; C-MPO = maintaining a positive outlook of caregivers; C-Strength = strength of caregivers; C-Tenacity = tenacity of caregivers; C-Optimism = optimism of caregivers; P-AR = acceptance-resignation of patients; P-Education = education level of patients; P-Avoidance = avoidance of patients.

The centrality index of the network nodes is shown in Supplementary Figure S16. The centrality of family communication and problem-solving was the highest among the dimensions of family resilience (Spouse r_S_ = 1.15, Offspring r_S_ = 1.50). The centrality of family communication and problem-solving among spousal caregivers was significantly lower than that among offspring caregivers (*p* = 0.042). Network accuracy and stability are shown in Supplementary Figures S17–S20.

## 4 Discussion

The aim of this study was to explore the status and influencing factors of family resilience among patients with COPD and caregivers and construct a related structural model and system of influencing factors for family resilience based on network analysis methods. We found that family communication and problem-solving play an important role in COPD families and improving the family concern of caregivers is related to the enhancement of family communication and problem-solving abilities.

### 4.1 Family communication and problem-solving play an important role in COPD families

The results indicated that, in the network model of family resilience and its influencing factors among patients with COPD, family communication and problem-solving were the central nodes, consistent with findings from a network analysis of pulmonary hypertension patients’ family resilience (Liu et al., 2024), highlighting their key role in the network. In both the COPD family resilience dimension network and the model of caregivers’ family resilience and its influencing factors, family communication and problem-solving emerged as central nodes, with significantly higher centrality among offspring caregivers than among spousal caregivers. Thus, interventions aimed at enhancing offspring caregivers’ family resilience should prioritize strengthening these abilities.

Communication is a key form of emotional support (Waldenburger et al., 2020), and the communicative theory of resilience (CTR) emphasizes that resilience is built and sustained through communication (Chen et al., 2022). Conversely, information gaps create uncertainty in family relationships (Abulaiti et al., 2022), while caregivers’ inappropriate emotional expression or poor coping skills in supporting patients may exacerbate disease recurrence. Families with strong communication skills openly share thoughts, feelings, and concerns; increase the frequency and depth of mutual expression between patients and caregivers; share each other’s doubts, wishes, and concerns; alleviate negative experiences; mobilize their own motivation; and make effective adjustments to improve family resilience. Problem-solving is task-oriented: Caregivers who adopt such strategies actively seek disease-related information and proper care methods (Bove et al., 2016; Waldenburger et al., 2020), accumulate professional knowledge about COPD in their daily lives, and strive to become a resource for coping with the disease (Hynes et al., 2012), reducing confusion during acute episodes. Ultimately, effective family communication and problem-solving abilities foster ongoing interaction among family members during caregiving, promoting mutual understanding and support to jointly tackle current challenges (Zhao et al., 2024).

In the COPD family resilience dimension network, the ability of family communication and problem-solving showed the strongest correlation with maintaining a positive outlook. Many patients lose confidence in treatment and rehabilitation due to the prolonged and recurrent nature of COPD, high treatment costs, and frequent relapses (Yin and Gu, 2021). When facing the disease, they are prone to feelings of despair, pessimism, and depression, becoming irritable and self-centered (Friedberg and Malefakis, 2022). They are unable or unwilling to contribute to the family, which increases the burden on caregivers, gradually diminishes the intimacy and love between spouses, weakens the initial caregiving motivation, and makes spousal caregivers feel unfairly treated due to their one-sided efforts. Therefore, caregiving gradually transforms into a responsibility and obligation imposed by marriage vows and social norms (Walsh, 2016). Communication gives patients and caregivers the opportunity to share, allowing family members to openly discuss and agree on decisions and solutions. Effective communication can reduce the patient’s self-perceived burden and alleviate caregivers’ anxiety, thereby fostering a sense of security, reducing the burden (Oeki and Takase, 2020), and shifting adverse perceptions. Maintaining a positive outlook toward the disease also motivates patients and caregivers to communicate with each other and adopt various coping styles to solve problems (Liu et al., 2024). Therefore, patients and caregivers should be guided to actively face the disease. Providing communication skills training to increase emotional expression and supportive listening between patients and spousal caregivers can help them better express themselves and listen to each other, achieving a state of harmony and balance. This approach can improve negative perceptions and maintain a positive and optimistic attitude.

In the network of family resilience dimensions among COPD families, the dimension of family communication and problem-solving between COPD patients and caregivers showed the strongest correlation, indicating that family communication and problem-solving between patients and caregivers influence each other. In addition to respiratory function limitation and decreased mobility, COPD patients are more prone to sleep disorders and psychological distress (Wang et al., 2023) and even personality changes, leading to negative self-concept, resistance to treatment, unwillingness to participate in daily activities, and avoidant/submissive behaviors such as failing to appreciate or be grateful for caregivers’ efforts. These behaviors not only reduce disease control but also diminish caregivers’ motivation for care and affection (Simpson et al., 2010), accelerating the transformation and breakdown of intimate relationships between patients and caregivers. Blame, control, and overprotection between patients and caregivers may also have adverse effects. Some spousal caregivers attribute COPD to patients’ smoking habits and criticize them for their perceived lack of willpower to quit smoking (Gabriel et al., 2014). Regarding COPD as a disease caused by self-behaviors, this perception can strain the relationship between patients and spousal caregivers and make it more difficult for caregivers to accept care responsibilities (Gabriel et al., 2014). Caregivers who feel burdened by caregiving are more inclined to underestimate the patient’s current ability to live and overprotect them. This not only causes caregivers to assume excessive family responsibilities and lose their self-identity, having adverse effects on physical and mental health and quality of life, but also reduces the patient’s sense of self-worth, which is unfavorable for disease coping (Meier et al., 2012). Therefore, the communication patterns regarding needs, perceptions, and provided support between patients and caregivers should be improved to help them openly and frankly exchange their feelings and needs. It is necessary to clarify the division of family responsibilities, jointly assume family responsibilities, respect patients’ sense of independence while ensuring their safety, encourage patients to undertake household chores appropriate to their physical condition to avoid overprotection, and maintain reciprocity and balance in intimate relationships.

FCPS19 was the central node in the network model of COPD family communication and problem-solving item associations. FCPS19 and FCPS20 showed the strongest correlation, indicating that it is of vital significance for both patients and caregivers to reach satisfactory solutions by discussing the encountered problems. However, among caregivers who desired more interaction, 83.1% of patients expressed no desire for communication, whereas among patients who desired more interaction, 66.7% of caregivers showed no preference for additional interaction (Fried et al., 2005). Significant discrepancies emerged between COPD patients and caregivers in their perceptions of disease progression and the appropriate timing for seeking medical assistance. Patients often do not express their true feelings and hope to delay or avoid treatment, while caregivers are highly alert to the basic changes in the condition and expect an early diagnosis and the initiation of treatment to control the symptoms. The difficulty in resolving such disagreements can further deteriorate the relationship (Suresh et al., 2022). Therefore, patients and caregivers should be guided to voice their concerns and doubts, so as to promote agreement regarding treatment decisions. Caregivers can encourage patients to actively seek treatment through suggestions and should avoid quarrels and conflicts caused by excessive urging, which may lead to negative consequences such as patients becoming emotionally agitated and experiencing breathing difficulties. It is necessary to maintain a balance between promoting patients’ treatment and respecting their autonomy.

The results of the network comparison showed that there was a statistically significant difference in the intensity of FCPS8 between the networks of patients and caregivers. Caregivers need explanations and clarifications from family members more than patients when they do not understand something, and unmet communication needs may add to the caregiver’s burden (Fried et al., 2005). During the long-term family caregiving process for chronic diseases, caregivers face various communication challenges, such as difficulty understanding patients’ needs and conveying key information to other family members (Wang, 2024). To avoid affecting the work and lives of other family members, caregivers may also reduce communication about the illness within the family. They may no longer confide in family members about their inner pain and burdens or seek their help. As time goes by, the illness becomes a topic that the whole family collectively avoids (Strang et al., 2018). Therefore, the communication mode should be actively transformed. Caregivers should work together with other family members to solve problems, seek external support, and relieve negative emotions.

### 4.2 Improving the family concern of caregivers is related to the enhancement of family communication and problem-solving abilities

In the network model of family resilience and its influencing factors among COPD patients, family communication and problem-solving of patients showed the strongest correlation with caregivers’ family concern. The higher the caregiver’s family concern, the higher the patient’s family communication and problem-solving level. When family members provide little support to caregivers, caregivers assume almost all of the caregiving responsibilities. With limited time to engage in other tasks, caregivers bear heavy physical, psychological, and social pressures. As a result, it is difficult for caregivers to actively participate in the patient’s treatment and rehabilitation (Sami et al., 2023), and they may provide less encouragement and support to patients (Zhang, 2020). A high level of family concern can provide a supportive family environment for caregivers, enabling them to become more actively involved in family caregiving and thereby strengthening the family resilience of patients. Family concern may help alleviate patients’ negative cognitions and adverse emotions by enhancing caregivers’ family concern, encouraging families to engage in open communication, and enabling caregivers to discuss and share the caregiving burden with patients and other family members. These processes can facilitate mutual understanding and effective interactions, help patients adapt positively to their roles, and reduce treatment avoidance stemming from shame, fear, and other undesirable emotions. Consequently, patients may receive greater physical care and emotional support and be able to actively utilize support resources to cope with stress (Mao et al., 2021).

In the network model of spousal caregivers’ family resilience and its influencing factors, family communication and problem-solving showed the strongest association with family concern among caregivers. The higher the family concern of spousal caregivers, the higher the level of family communication and problem-solving. Among the COPD patients and caregivers included in this study, the proportion of older adults was 67.0%. The physical functioning of spousal caregivers may gradually decline with age, accompanied by reduced cognitive and disease-related understanding. They may have limited ability to use online resources to collect relevant information (Jiang, 2020). Spousal caregivers have a close relationship with patients and show deep emotional dependence, making it difficult for them to accept changes in the patients’ physical condition. They face high mental stress when dealing with patients and lack confidence in completing care tasks (Tian, 2023). Therefore, medical staff should provide regular education on disease knowledge and care for COPD families, encouraging family members to communicate openly. This may help spousal caregivers acquire a more scientific understanding of daily care practices and appropriate rehabilitation exercises. It is also necessary to encourage family members to provide greater care and assistance to spousal caregivers, create a relaxed family communication atmosphere, and provide emotional support through caring, companionship, listening, and encouragement. These measures may help enhance the family communication and problem-solving abilities of spousal caregivers.

In the network model of offspring caregivers’ family resilience and its influencing factors, higher levels of family care were associated with better family communication and problem-solving. Offspring caregivers balance family, social, and work roles while caring for patients, facing multiple pressures. In addition, they often have higher education levels, making it harder to accept losing independence and autonomy (Gullick, 2012). Long-term parental care isolates them from other family and social activities; they may feel sad or angry over lost relationships, roles, and identities (Simpson et al., 2010). Furthermore, lacking basic knowledge of disease progression and symptom management erodes their confidence, fostering isolation and powerlessness (Simpson et al., 2010; Suresh et al., 2022). As an emotional bond for family members, the family provides strong spiritual support. When offspring caregivers’ stress exceeds their coping capacity, families should increase care and assistance—offering emotional support via companionship, listening, and encouragement. Family members should also share care responsibilities, use resources to solve problems, and build social support networks to relieve offspring caregivers’ work and life pressures. This enables caregivers to leverage the family system’s strengths, thereby enhancing their family communication and problem-solving abilities.

## 5 Limitations

This study has certain limitations. First, as a cross-sectional study conducted at a single time point, this study cannot establish the temporal sequence of the variables and does not allow for causal inferences. It is unable to determine the development trends of family resilience among COPD patients and caregivers, as well as the mutual influence relationships and directions between the network central nodes and other nodes of the influencing factors. Second, the sample size in this study was small, and the types of COPD patients and caregivers were not further categorized. Third, due to limitations in time, location, and human resources, all participants in this study were recruited from only two hospitals in Tianjin. The limited representativeness of the sample may restrict the generalizability of the study findings.

## 6 Conclusion

Interventions aimed at improving family communication and problem-solving skills can enhance family resilience among patients and caregivers, especially offspring caregivers. Both patients and caregivers prefer to discuss problems with family members to find satisfactory solutions. Compared to patients, caregivers require more explanations from family members when they do not understand something. Nurses should encourage other family members to care more for caregivers, monitor changes in family communication and problem-solving between patients and caregivers, and explore their potential connections. Future studies should adopt longitudinal designs to clarify the causal relationships among variables. In addition, larger-scale, multicenter studies are needed to deeply explore subgroup characteristics across different disease stages and caregiver types, as well as to further develop and validate targeted family intervention programs based on network-identified targets.

## Data Availability

The raw data supporting the conclusions of this article will be made available by the authors, without undue reservation.
