# Supplementary material for: Profiling family resilience in chronic obstructive pulmonary disease: a network analysis approach to identifying intervention targets
Source: Front Psychol. 2026 Oct 6;17:1932841. doi: 10.3389/fpsyg.2026.1932841 (PMC13680222; doi:10.3389/fpsyg.2026.1932841)
Supplement: Supplementary file 1 [file Supplementary_file_1.docx]

**Supplementary material**

Table S1 The edge weights of the family resilience dimension association network (n=221)

|  | 1 | 2 | 3 | 4 | 5 | 6 |
| --- | --- | --- | --- | --- | --- | --- |
| 1.P-FCPS |  |  |  |  |  |  |
| 2.P-USR | 0.13 |  |  |  |  |  |
| 3.P-MPO | 0.47 | 0.05 |  |  |  |  |
| 4.C-FCPS | 0.36 | 0.10 | 0.16 |  |  |  |
| 5.C-USR | 0.03 | 0.20 | 0 | 0.18 |  |  |
| 6.C-MPO | 0.10 | 0 | 0.04 | 0.53 | 0.17 |  |

P-FCPS= family communication and problem solving of patients；P-USR=utilizing social resources of patients；P-MPO= maintaining a positive outlook of patients；C-FCPS= family communication and problem solving of caregivers；C-USR= utilizing social resources of caregivers；C-MPO= maintaining a positive outlook of caregivers

Figure S1 Centrality index of family resilience dimension association network (n=221)

Figure S2 Bootstrapped difference test node strength (n=221).

Figure S3 Bootstrapped difference test edges (n=221).

Figure S4 Bootstrapped confidence intervals of estimated edge-weights for the estimated network (n=221).

Figure S5 Stability of centrality indices by case dropping subset bootstrap (n=221).

|  | 1 | 2 | 3 | 4 | 5 | 8 | 9 | 10 | 11 | 12 | 15 | 16 | 17 | 18 | 19 | 20 | 21 | 22 | 27 | 29 | 30 | 31 | 32 |
| --- | --- | --- | --- | --- | --- | --- | --- | --- | --- | --- | --- | --- | --- | --- | --- | --- | --- | --- | --- | --- | --- | --- | --- |
| 1 |  |  |  |  |  |  |  |  |  |  |  |  |  |  |  |  |  |  |  |  |  |  |  |
| 2 | 0.14 |  |  |  |  |  |  |  |  |  |  |  |  |  |  |  |  |  |  |  |  |  |  |
| 3 | 0.04 | 0.32 |  |  |  |  |  |  |  |  |  |  |  |  |  |  |  |  |  |  |  |  |  |
| 4 | 0.01 | 0 | 0.23 |  |  |  |  |  |  |  |  |  |  |  |  |  |  |  |  |  |  |  |  |
| 5 | 0.12 | 0.05 | 0 | 0.27 |  |  |  |  |  |  |  |  |  |  |  |  |  |  |  |  |  |  |  |
| 8 | 0.02 | 0.002 | 0 | 0 | 0.06 |  |  |  |  |  |  |  |  |  |  |  |  |  |  |  |  |  |  |
| 9 | 0.05 | 0.02 | 0.01 | 0 | 0.07 | 0 |  |  |  |  |  |  |  |  |  |  |  |  |  |  |  |  |  |
| 10 | 0.01 | 0 | 0.04 | 0 | 0.01 | 0.01 | 0.24 |  |  |  |  |  |  |  |  |  |  |  |  |  |  |  |  |
| 11 | 0 | 0 | 0 | 0 | 0.06 | 0 | 0.07 | 0.21 |  |  |  |  |  |  |  |  |  |  |  |  |  |  |  |
| 12 | 0.02 | 0 | 0 | 0.13 | 0 | 0.05 | 0.03 | 0 | 0.34 |  |  |  |  |  |  |  |  |  |  |  |  |  |  |
| 15 | 0.04 | 0.03 | 0.17 | 0.02 | 0 | 0.13 | 0 | 0.02 | 0.05 | 0.07 |  |  |  |  |  |  |  |  |  |  |  |  |  |
| 16 | 0.04 | 0 | 0 | 0 | 0.11 | 0.05 | 0 | 0.01 | 0 | 0 | 0.21 |  |  |  |  |  |  |  |  |  |  |  |  |
| 17 | 0 | 0 | 0.04 | 0.06 | 0.04 | 0 | 0 | 0 | 0.04 | 0.03 | 0 | 0.20 |  |  |  |  |  |  |  |  |  |  |  |
| 18 | 0 | 0 | 0.02 | 0.08 | 0 | 0.12 | 0 | 0 | 0.06 | 0 | 0 | 0 | 0.33 |  |  |  |  |  |  |  |  |  |  |
| 19 | 0 | 0.08 | 0 | 0.01 | 0 | 0.05 | 0 | 0 | 0.02 | 0.05 | 0.04 | 0 | 0.06 | 0 |  |  |  |  |  |  |  |  |  |
| 20 | 0.08 | 0.04 | 0.03 | 0 | 0 | 0.06 | 0.04 | 0 | 0 | 0.02 | 0 | 0 | 0.01 | 0 | 0.53 |  |  |  |  |  |  |  |  |
| 21 | 0.003 | 0 | 0 | 0 | 0 | 0 | 0 | 0 | 0 | 0.02 | 0 | 0 | 0 | 0.22 | 0.04 | 0.16 |  |  |  |  |  |  |  |
| 22 | 0 | 0 | 0 | 0.04 | 0 | 0 | 0.12 | 0.04 | 0.04 | 0.01 | 0.01 | 0.02 | 0 | 0.005 | 0.02 | 0.01 | 0.36 |  |  |  |  |  |  |
| 27 | 0 | 0 | 0 | 0.03 | 0 | 0.08 | 0.04 | 0.04 | 0.04 | 0.14 | 0.16 | 0.03 | 0 | 0.07 | 0.03 | 0 | 0 | 0 |  |  |  |  |  |
| 28 | 0.02 | 0 | 0 | 0.03 | 0.02 | 0 | 0 | 0 | 0 | 0.03 | 0.01 | 0.09 | 0 | 0.04 | 0 | 0 | 0.14 | 0 | 0.25 |  |  |  |  |
| 30 | 0 | 0 | 0.16 | 0.01 | 0.02 | 0.01 | 0.004 | 0 | 0 | 0 | 0.02 | 0.11 | 0 | 0.04 | 0.09 | 0.04 | 0 | 0.06 | 0.04 | 0.03 |  |  |  |
| 31 | 0.02 | 0.11 | 0 | 0 | 0.08 | 0 | 0.12 | 0 | 0 | 0 | 0 | 0 | 0.04 | 0.09 | 0.05 | 0 | 0.02 | 0.02 | 0.06 | 0 | 0.17 |  |  |
| 32 | 0.05 | 0.10 | 0.05 | 0.07 | 0 | 0 | 0 | 0 | 0.03 | 0 | 0.04 | 0 | 0.04 | 0.04 | 0.05 | 0.02 | 0 | 0 | 0 | 0.14 | 0.04 | 0.31 |  |

Table S2 Family communication and problem solving item network edge weights of patients

|  | 1 | 2 | 3 | 4 | 5 | 8 | 9 | 10 | 11 | 12 | 15 | 16 | 17 | 18 | 19 | 20 | 21 | 22 | 27 | 29 | 30 | 31 | 32 |
| --- | --- | --- | --- | --- | --- | --- | --- | --- | --- | --- | --- | --- | --- | --- | --- | --- | --- | --- | --- | --- | --- | --- | --- |
| 1 |  |  |  |  |  |  |  |  |  |  |  |  |  |  |  |  |  |  |  |  |  |  |  |
| 2 | 0.10 |  |  |  |  |  |  |  |  |  |  |  |  |  |  |  |  |  |  |  |  |  |  |
| 3 | 0.18 | 0.24 |  |  |  |  |  |  |  |  |  |  |  |  |  |  |  |  |  |  |  |  |  |
| 4 | 0.09 | 0.12 | 0.23 |  |  |  |  |  |  |  |  |  |  |  |  |  |  |  |  |  |  |  |  |
| 5 | 0.01 | 0 | 0 | 0.25 |  |  |  |  |  |  |  |  |  |  |  |  |  |  |  |  |  |  |  |
| 8 | 0 | 0.09 | 0 | 0 | 0.04 |  |  |  |  |  |  |  |  |  |  |  |  |  |  |  |  |  |  |
| 9 | 0.10 | 0 | 0.04 | 0 | 0.10 | 0.16 |  |  |  |  |  |  |  |  |  |  |  |  |  |  |  |  |  |
| 10 | 0.01 | 0.002 | 0.02 | 0 | 0 | 0 | 0.16 |  |  |  |  |  |  |  |  |  |  |  |  |  |  |  |  |
| 11 | 0 | 0.07 | 0 | 0 | 0 | 0 | 0 | 0.19 |  |  |  |  |  |  |  |  |  |  |  |  |  |  |  |
| 12 | 0.05 | 0 | 0.04 | 0 | 0.07 | 0 | 0 | 0 | 0.31 |  |  |  |  |  |  |  |  |  |  |  |  |  |  |
| 15 | 0.14 | 0.01 | 0.05 | 0 | 0 | 0.20 | 0 | 0.15 | 0 | 0.09 |  |  |  |  |  |  |  |  |  |  |  |  |  |
| 16 | 0 | 0.004 | 0 | 0.02 | 0 | 0 | 0 | 0 | 0 | 0 | 0.13 |  |  |  |  |  |  |  |  |  |  |  |  |
| 17 | 0 | 0 | 0 | 0.01 | 0.14 | 0 | 0 | 0 | 0.09 | 0 | 0 | 0.15 |  |  |  |  |  |  |  |  |  |  |  |
| 18 | 0.05 | 0 | 0 | 0.06 | 0 | 0.06 | 0.15 | 0.004 | 0.04 | 0.04 | 0 | 0.03 | 0.27 |  |  |  |  |  |  |  |  |  |  |
| 19 | 0.05 | 0.08 | 0.05 | 0.08 | 0 | 0 | 0 | 0.06 | 0 | 0.05 | 0.03 | 0 | 0 | 0.17 |  |  |  |  |  |  |  |  |  |
| 20 | 0.02 | 0 | 0.02 | 0.01 | 0 | 0.14 | 0.01 | 0.01 | 0 | 0 | 0.01 | 0.07 | 0.03 | 0 | 0.37 |  |  |  |  |  |  |  |  |
| 21 | 0.03 | 0.04 | 0 | 0 | 0.07 | 0 | 0 | 0 | 0.05 | 0.02 | 0.07 | 0 | 0 | 0 | 0 | 0.10 |  |  |  |  |  |  |  |
| 22 | 0 | 0 | 0.05 | 0.02 | 0 | 0.02 | 0.03 | 0 | 0.13 | 0 | 0 | 0 | 0.05 | 0.01 | 0 | 0.08 | 0.33 |  |  |  |  |  |  |
| 27 | 0.01 | 0 | 0.04 | 0 | 0 | 0.17 | 0.04 | 0.02 | 0.03 | 0.08 | 0.08 | 0.12 | 0 | 0 | 0.09 | 0.01 | 0 | 0.01 |  |  |  |  |  |
| 28 | 0.02 | 0 | 0 | 0.12 | 0.02 | 0.05 | 0 | 0.08 | 0 | 0.02 | 0 | 0.07 | 0.18 | 0 | 0.04 | 0 | 0.08 | 0.10 | 0.07 |  |  |  |  |
| 30 | 0 | 0.12 | 0.03 | 0.03 | 0 | 0 | 0 | 0 | 0 | 0.08 | 0.04 | 0.02 | 0.06 | 0.01 | 0 | 0.04 | 0.06 | 0 | 0.13 | 0.01 |  |  |  |
| 31 | 0 | 0 | 0.03 | 0 | 0.06 | 0.10 | 0 | 0 | 0.004 | 0.004 | 0 | 0.05 | 0 | 0.12 | 0.07 | 0.05 | 0.03 | 0 | 0.11 | 0 | 0.14 |  |  |
| 32 | 0 | 0 | 0.04 | 0 | 0.09 | 0.02 | 0.03 | 0 | 0.14 | 0 | 0 | 0.08 | 0 | 0.04 | 0.06 | 0 | 0 | 0.01 | 0 | 0.09 | 0.08 | 0.25 |  |

Table S3 Family communication and problem solving item network edge weights of caregivers

 Table S family communication and problem solving item network edge weights of patient

Figure S6 Comparison of centrality index of family resilience item association network among patients and caregivers (n=221).

a patients

b caregivers

Figure S7 Bootstrapped difference test node strength (n=221).

a patients

b caregivers

Figure S8 Bootstrapped difference test edges (n=221).

a patients

b caregivers

Figure S9 Bootstrapped confidence intervals of estimated edge-weights for the estimated network (n=221).

a patients

b caregivers

Figure S10 Stability of centrality indices by case dropping subset bootstrap (n=221).

Table S4 Comparison of family resilience in COPD patients between patient and caregiver general factors (n =221).

|  | Variables | N(%) | Professional  identity *M(SD)* | *t/F* | *p* |
| --- | --- | --- | --- | --- | --- |
| Patient | Gender |  |  | -0.550 | 0.583 |
|  | Male | 160(72.4) | 94.86±10.29 |  |  |
|  | Female | 61(27.6) | 95.70±9.87 |  |  |
|  | Age |  |  | 4.211 | 0.016 |
|  | ≤60 | 25(11.3) | 100.48±8.71 |  |  |
|  | 61~74 | 142(64.3) | 94.64±9.43 |  |  |
|  | ≥75 | 54(24.4) | 93.80±11.92 |  |  |
|  | Education level |  |  | 17.748 | <0.001 |
|  | Primary School or Below | 45(20.4) | 90.18±6.83 |  |  |
|  | Junior school | 116(52.5) | 93.44±9.71 |  |  |
|  | Senior high school/Technical secondary school | 43(19.4) | 100.77±9.33 |  |  |
|  | College and above | 17(7.7) | 105.06±10.31 |  |  |
|  | Marital status |  |  | 2.965 | 0.006 |
|  | Married | 197(89.1) | 96.01±9.33 |  |  |
|  | Divorced/ Widowed/ Spinsterhood | 24(10.9) | 87.63±13.46 |  |  |
|  | Occupation |  |  | 20.645 | <0.001 |
|  | In-job training | 15(6.8) | 96.00±14.28 |  |  |
|  | Retirement | 163(73.8) | 97.18±8.95 |  |  |
|  | Unemployed | 19.4(43) | 86.88±8.83 |  |  |
|  | COPD severity  Mild  Moderate  Severe or extremely severe |  |  | 29.123 | <0.001 |
|  |  | 15(6.8) | 103.20±7.50 |  |  |
|  |  | 96(43.4) | 99.06±7.90 |  |  |
|  |  | 110(49.8) | 90.53±10.15 |  |  |
|  | Duration of COPD disease |  |  | 7.180 | 0.001 |
|  | <1 year | 30(13.6) | 96.60±7.82 |  |  |
|  | 1-5 years | 61(27.6) | 98.70±9.91 |  |  |
|  | >5 years | 130(58.8) | 93.05±10.29 |  |  |
|  | Number of complications |  |  | 12.031 | <0.001 |
|  | 0 | 37(16.7) | 100.70±8.69 |  |  |
|  | 1 | 81(36.7) | 96.41±8.61 |  |  |
|  | ≥2 | 103(46.6) | 92.05±10.76 |  |  |
|  | Smoking status |  |  | 4.971 | 0.008 |
|  | Never smoked | 52(23.5) | 97.85±9.71 |  |  |
|  | Quitting smoking | 112(50.7) | 95.45±10.00 |  |  |
|  | Not quitting smoking | 57(25.8) | 91.89±10.20 |  |  |
|  | Number of exacerbations in the past year |  |  | 15.116 | <0.001 |
|  | 0 | 127(57.5) | 97.35±8.63 |  |  |
|  | 1 | 69(31.2) | 94.23±11.05 |  |  |
|  | ≥2 | 25(11.3) | 86.00±9.70 |  |  |
|  | Medical payment method |  |  | 6.340 | <0.001 |
|  | Medical insurance | 170(76.9) | 97.28±9.50 |  |  |
|  | New rural cooperative medical | 51(23.1) | 87.80±8.90 |  |  |
|  | Number of offspring |  |  | 8.338 | <0.001 |
|  | 0 | 5(2.3) | 74.20±10.80 |  |  |
|  | 1 | 111(50.2) | 96.14±10.33 |  |  |
|  | 2 | 77(34.8) | 95.36±9.23 |  |  |
|  | ≥3 | 28(12.7) | 93.93±7.91 |  |  |
|  | Family atmosphere |  |  | 5.540 | <0.001 |
|  | Harmony | 195(88.2) | 96.77±8.49 |  |  |
|  | General or tense | 26(11.8) | 82.50±12.77 |  |  |
|  | Per capita monthly household income |  |  | 64.508 | <0.001 |
|  | ≤1000 | 9(4.1) | 76.56±7.96 |  |  |
|  | 1001~3000 | 58(26.2) | 88.05±7.37 |  |  |
|  | 3001~5000 | 94(42.5) | 95.55±7.54 |  |  |
|  | ≥5001 | 60(27.2) | 103.97±7.28 |  |  |
| Caregiver | Gender |  |  | -1.133 | 0.260 |
|  | Male | 62(28.1) | 93.69±12.26 |  |  |
|  | Female | 159(71.9) | 95.64±9.21 |  |  |
|  | Age |  |  | -0.113 | 0.910 |
|  | ≤60 | 121(54.8) | 95.02±10.05 |  |  |
|  | >60 | 100(45.2) | 95.18±10.35 |  |  |
|  | Education level |  |  | 17.208 | <0.001 |
|  | Primary School or Below | 13(5.9) | 87.54±11.61 |  |  |
|  | Junior school | 89(40.3) | 91.58±9.09 |  |  |
|  | Senior high school/ Technical secondary school | 60(27.1) | 95.52±8.21 |  |  |
|  | College and above | 59(26.7) | 101.63±9.73 |  |  |
|  | Marital status |  |  | 4.714 | <0.001 |
|  | Married | 192(86.9) | 96.48±9.20 |  |  |
|  | Divorced/ Widowed/ Spinsterhood | 29(13.1) | 89.90±11.56 |  |  |
|  | Occupation |  |  | 5.769 | 0.004 |
|  | In-job training | 93(42.1) | 95.54±9.92 |  |  |
|  | Retirement | 93(42.1) | 96.58±10.26 |  |  |
|  | Unemployed | 35(15.8) | 89.97±9.15 |  |  |
|  | Relationship with patients |  |  | 6.380 | 0.004 |
|  | Spouse | 101(45.7) | 96.94±9.52 |  |  |
|  | Offspring | 103(46.6) | 94.90±9.35 |  |  |
|  | Other | 17(7.7) | 85.29±13.16 |  |  |
|  | Chronic disease status |  |  | -4.135 | <0.001 |
|  | Yes | 83(37.6) | 91.37±11.23 |  |  |
|  | No | 138(62.4) | 97.33±8.77 |  |  |
|  | Daily length of care |  |  | 10.946 | <0.001 |
|  | Less than 6 hours | 52(23.5) | 101.02±9.57 |  |  |
|  | 6-12 hours | 56(25.4) | 95.95±10.47 |  |  |
|  | 12-18 hours | 40(18.1) | 91.80±8.69 |  |  |
|  | More than 18 hours | 73(33.0) | 92.03±9.20 |  |  |
|  | Duration of care |  |  | 25.734 | <0.001 |
|  | Within one year | 132(59.7) | 99.44±7.48 |  |  |
|  | 1-3 years | 35(15.9) | 90.14±10.32 |  |  |
|  | 3-5 years | 25(11.3) | 90.16±6.84 |  |  |
|  | More than 5 years | 29(13.1) | 85.55±12.04 |  |  |
|  | The main householder |  |  | -6.022 | <0.001 |
|  | Yes | 137(62.0) | 92.10±9.89 |  |  |
|  | No | 84(38.0) | 99.98±8.63 |  |  |
|  | Family breadwinner |  |  | -5.256 | <0.001 |
|  | Yes | 66(29.9) | 89.89±10.88 |  |  |
|  | No | 155(70.1) | 97.31±9.00 |  |  |

Table S5 Comparison of family resilience in caregivers of COPD patients between patient and caregiver general factors (N =221).

|  | Variables | N(%) | Professional  identity *M(SD)* | *t/F* | *p* |
| --- | --- | --- | --- | --- | --- |
| Patient | Gender |  |  | 0.799 | 0.425 |
|  | Male | 160(72.4) | 97.69±11.35 |  |  |
|  | Female | 61(27.6) | 96.33±11.37 |  |  |
|  | Age |  |  | 2.832 | 0.061 |
|  | ≤60 | 25(11.3) | 102.28±10.46 |  |  |
|  | 61~74 | 142(64.3) | 96.89±11.04 |  |  |
|  | ≥75 | 54(24.4) | 96.13±12.13 |  |  |
|  | Education level |  |  | 26.718 | <0.001 |
|  | Primary School or Below | 45(20.4) | 90.51±7.33 |  |  |
|  | Junior school | 116(52.5) | 95.33±10.30 |  |  |
|  | Senior high school/Technical secondary school | 43(19.4) | 104.88±9.78 |  |  |
|  | College and above | 17(7.7) | 109.76±11.49 |  |  |
|  | Marital status |  |  | 3.884 | <0.001 |
|  | Married | 197(89.1) | 98.32±10.80 |  |  |
|  | Divorced/ Widowed/ Spinsterhood | 24(10.9) | 89.08±12.59 |  |  |
|  | Occupation |  |  | 27.544 | <0.001 |
|  | In-job training | 15(6.8) | 96.20±13.74 |  |  |
|  | Retirement | 163(73.8) | 100.10±10.24 |  |  |
|  | Unemployed | 19.4(43) | 87.16±8.43 |  |  |
|  | COPD severity  Mild  Moderate  Severe or extremely severe |  |  | 22.266 | <0.001 |
|  |  | 15(6.8) | 104.87±6.76 |  |  |
|  |  | 96(43.4) | 101.42±9.51 |  |  |
|  |  | 110(49.8) | 92.71±11.45 |  |  |
|  | Duration of COPD disease |  |  | 7.579 | 0.001 |
|  | <1 year | 30(13.6) | 99.73±9.01 |  |  |
|  | 1-5 years | 61(27.6) | 101.21±10.52 |  |  |
|  | >5 years | 130(58.8) | 94.93±11.65 |  |  |
|  | Number of complications |  |  | 17.885 | <0.001 |
|  | 0 | 37(16.7) | 105.11±10.91 |  |  |
|  | 1 | 81(36.7) | 98.74±9.25 |  |  |
|  | ≥2 | 103(46.6) | 93.40±11.38 |  |  |
|  | Smoking status |  |  | 3.104 | 0.047 |
|  | Never smoked | 52(23.5) | 99.21±10.91 |  |  |
|  | Quitting smoking | 112(50.7) | 98.01±11.91 |  |  |
|  | Not quitting smoking | 57(25.8) | 94.23±10.10 |  |  |
|  | Number of exacerbations in the past year |  |  | 15.103 | <0.001 |
|  | 0 | 127(57.5) | 99.67±10.32 |  |  |
|  | 1 | 69(31.2) | 96.77±11.65 |  |  |
|  | ≥2 | 25(11.3) | 86.88±9.62 |  |  |
|  | Medical payment method |  |  | 28.090 | <0.001 |
|  | Medical insurance | 170(76.9) | 99.92±10.63 |  |  |
|  | New rural cooperative medical | 51(23.1) | 90.20±8.41 |  |  |
|  | Number of offspring |  |  | 6.781 | <0.001 |
|  | 0 | 5(2.3) | 77.20±7.60 |  |  |
|  | 1 | 111(50.2) | 98.95±11.60 |  |  |
|  | 2 | 77(34.8) | 96.96±10.87 |  |  |
|  | ≥3 | 28(12.7) | 95.43±8.36 |  |  |
|  | Family atmosphere |  |  | 7.164 | <0.001 |
|  | Harmony | 195(88.2) | 99.12±10.32 |  |  |
|  | General or tense | 26(11.8) | 83.81±9.58 |  |  |
|  | Per capita monthly household income |  |  | 68.919 | <0.001 |
|  | ≤1000 | 9(4.1) | 78.78±5.04 |  |  |
|  | 1001~3000 | 58(26.2) | 88.16±7.30 |  |  |
|  | 3001~5000 | 94(42.5) | 98.51±8.33 |  |  |
|  | ≥5001 | 60(27.2) | 107.08±9.03 |  |  |
| Caregiver | Gender |  |  | -2.423 | 0.016 |
|  | Male | 62(28.1) | 94.39±10.68 |  |  |
|  | Female | 159(71.9) | 98.46±11.43 |  |  |
|  | Age |  |  | 1.650 | 0.100 |
|  | ≤60 | 121(54.8) | 98.43±12.53 |  |  |
|  | >60 | 100(45.2) | 95.97±9.61 |  |  |
|  | Education level |  |  | 29.074 | <0.001 |
|  | Primary School or Below | 13(5.9) | 88.08±11.49 |  |  |
|  | Junior school | 89(40.3) | 92.60±8.75 |  |  |
|  | Senior high school/ Technical secondary school | 60(27.1) | 96.43±8.89 |  |  |
|  | College and above | 59(26.7) | 107.37±10.36 |  |  |
|  | Marital status |  |  | 3.211 | 0.003 |
|  | Married | 192(86.9) | 98.48±10.37 |  |  |
|  | Divorced/ Widowed/ Spinsterhood | 29(13.1) | 89.59±14.37 |  |  |
|  | Occupation |  |  | 7.282 | 0.001 |
|  | In-job training | 93(42.1) | 99.06±12.33 |  |  |
|  | Retirement | 93(42.1) | 97.99±9.54 |  |  |
|  | Unemployed | 35(15.8) | 90.89±11.11 |  |  |
|  | Relationship with patients |  |  | 6.380 | 0.004 |
|  | Spouse | 101(45.7) | 97.82±9.04 |  |  |
|  | Offspring | 103(46.6) | 98.27±12.23 |  |  |
|  | Other | 17(7.7) | 88.53±14.73 |  |  |
|  | Chronic disease status |  |  | -5.727 | <0.001 |
|  | Yes | 83(37.6) | 92.05±10.36 |  |  |
|  | No | 138(62.4) | 100.49±10.75 |  |  |
|  | Daily length of care |  |  | 25.482 | <0.001 |
|  | Less than 6 hours | 52(23.5) | 106.19±9.15 |  |  |
|  | 6-12 hours | 56(25.4) | 99.43±10.43 |  |  |
|  | 12-18 hours | 40(18.1) | 91.23±8.37 |  |  |
|  | More than 18 hours | 73(33.0) | 92.71±10.51 |  |  |
|  | Duration of care |  |  | 34.107 | <0.001 |
|  | Within one year | 132(59.7) | 102.27±9.24 |  |  |
|  | 1-3 years | 35(15.9) | 93.74±8.91 |  |  |
|  | 3-5 years | 25(11.3) | 89.60±7.66 |  |  |
|  | More than 5 years | 29(13.1) | 85.72±11.90 |  |  |
|  | The main householder |  |  | -6.854 | <0.001 |
|  | Yes | 137(62.0) | 93.59±10.50 |  |  |
|  | No | 84(38.0) | 103.39±10.02 |  |  |
|  | Family breadwinner |  |  | -5.963 | <0.001 |
|  | Yes | 66(29.9) | 90.83±11.42 |  |  |
|  | No | 155(70.1) | 100.08±10.16 |  |  |

Table S6 Correlations between family resilience and other factors (n=221)

|  | Patients | | | | | | | | Caregivers | | | | | | | |
| --- | --- | --- | --- | --- | --- | --- | --- | --- | --- | --- | --- | --- | --- | --- | --- | --- |
|  | 1 | 2 | 3 | 4 | 5 | 6 | 7 | 8 | 1 | 2 | 3 | 4 | 5 | 6 | 7 | 8 |
| 1 | 1 |  |  |  |  |  |  |  |  |  |  |  |  |  |  |  |
| 2 | 0.789*** | 1 |  |  |  |  |  |  |  |  |  |  |  |  |  |  |
| 3 | 0.719*** | 0.750*** | 1 |  |  |  |  |  |  |  |  |  |  |  |  |  |
| 4 | 0.715*** | 0.784*** | 0.753*** | 1 |  |  |  |  |  |  |  |  |  |  |  |  |
| 5 | 0.749*** | 0.722*** | 0.674*** | 0.726*** | 1 |  |  |  |  |  |  |  |  |  |  |  |
| 6 | 0.694*** | 0.716*** | 0.717*** | 0.721*** | 0.662*** | 1 |  |  |  |  |  |  |  |  |  |  |
| 7 | -0.625*** | -0.595*** | -0.583*** | -0.586*** | -0.603*** | -0.673*** | 1 |  |  |  |  |  |  |  |  |  |
| 8 | -0.735*** | -0.758*** | -0.730*** | -0.774*** | -0.786*** | -0.788*** | 0.685*** | 1 |  |  |  |  |  |  |  |  |
| 1 | 0.804*** | 0.683*** | 0.639*** | 0.622*** | 0.723*** | 0.686*** | -0.648*** | -0.723*** | 1 |  |  |  |  |  |  |  |
| 2 | 0.670*** | 0.686*** | 0.577*** | 0.591*** | 0.629*** | 0.636*** | -0.586*** | -0.628*** | 0.768*** | 1 |  |  |  |  |  |  |
| 3 | 0.648*** | 0.662*** | 0.693*** | 0.623*** | 0.605*** | 0.674*** | -0.592*** | -0.629*** | 0.718*** | 0.767*** | 1 |  |  |  |  |  |
| 4 | 0.657*** | 0.591*** | 0.586*** | 0.570*** | 0.638*** | 0.616*** | -0.585*** | -0.605*** | 0.740*** | 0.817*** | 0.713*** | 1 |  |  |  |  |
| 5 | 0.748*** | 0.697*** | 0.622*** | 0.664*** | 0.797*** | 0.671*** | -0.559*** | -0.720*** | 0.779*** | 0.701*** | 0.758*** | 0.714*** | 1 |  |  |  |
| 6 | 0.562*** | 0.600*** | 0.551*** | 0.554*** | 0.517*** | 0.625*** | -0.600*** | -0.563*** | 0.654*** | 0.715*** | 0.756*** | 0.681*** | 0.674*** | 1 |  |  |
| 7 | -0.464*** | -0.429*** | -0.340*** | -0.449*** | -0.450*** | -0.413*** | 0.391*** | 0.461*** | -0.430*** | -0.315*** | -0.401*** | -0.386*** | -0.554*** | -0.357*** | 1 |  |
| 8 | -0.634*** | -0.662*** | -0.512*** | -0.597*** | -0.659*** | -0.559*** | 0.537*** | 0.656*** | -0.663*** | -0.596*** | -0.563*** | -0.626*** | -0.766*** | -0.539*** | 0.538*** | 1 |

****P* < 0.00

Patients：1. Family resilience; 2. Resilience; 3. Perceived social support; 4. Self-efficacy for managing chronic disease; 5. Family concern; 6. Confrontation; 7. Avoidance; 8. Acceptance-resignation. Caregivers：1. Family resilience; 2. Resilience; 3. Perceived social support; 4. General self-efficacy; 5. Family concern；6. Positive coping; 7. Negative coping; 8. Caregiver burden.

Table S7 Family resilience and its influencing factors network edge weight in COPD patients (n=221)

|  | 1 | 2 | 3 | 4 | 5 | 6 | 7 | 8 | 9 | 10 | 11 | 12 |
| --- | --- | --- | --- | --- | --- | --- | --- | --- | --- | --- | --- | --- |
| 1.P-FCPS |  |  |  |  |  |  |  |  |  |  |  |  |
| 2.P-USR | 0.08 |  |  |  |  |  |  |  |  |  |  |  |
| 3.P-MPO | 0.38 | 0.04 |  |  |  |  |  |  |  |  |  |  |
| 4.Offspring | 0.05 | 0 | 0 |  |  |  |  |  |  |  |  |  |
| 5.Income | 0.22 | 0 | 0.06 | -0.03 |  |  |  |  |  |  |  |  |
| 6.P-Tenacity | 0.04 | 0.10 | 0 | -0.01 | 0.02 |  |  |  |  |  |  |  |
| 7.P-Strength | 0.11 | 0.07 | 0.12 | 0 | 0 | 0.53 |  |  |  |  |  |  |
| 8.P-Optimism | 0.07 | 0 | 0.02 | 0 | 0 | 0.20 | 0.06 |  |  |  |  |  |
| 9.P-Family Support | 0.19 | 0 | 0.06 | 0 | 0.15 | 0.08 | 0.01 | 0.04 |  |  |  |  |
| 10.P-Friend Support | 0.03 | 0 | 0 | -0.05 | 0 | 0.03 | 0 | 0.16 | 0.19 |  |  |  |
| 11.P-Other Support | 0.05 | 0.11 | 0 | 0 | -0.01 | 0.01 | 0.08 | 0.05 | 0 | 0.60 |  |  |
| 12.C-FC | 0.23 | 0.10 | 0.12 | -0.03 | 0.21 | 0 | 0.08 | 0.18 | 0.11 | 0 | 0 |  |

Figure S11 Centrality indices of family resilience and its influencing factors network among patients (n=221).

Figure S12 Bootstrapped difference test node strength (n=221).

Figure S13 Bootstrapped difference test edges (n=221).

Figure S14 Bootstrapped confidence intervals of estimated edge-weights for the estimated network (n=221).

Figure S15 Stability of centrality indices by case dropping subset bootstrap(n=221).

Table S8 Family resilience and its influencing factors network edge weight in spouse caregivers (n=101)

|  | 1 | 2 | 3 | 4 | 5 | 6 | 7 | 8 | 9 | 10 |
| --- | --- | --- | --- | --- | --- | --- | --- | --- | --- | --- |
| 1. C-FCPS |  |  |  |  |  |  |  |  |  |  |
| 2. C-USR | 0.04 |  |  |  |  |  |  |  |  |  |
| 3. C-MPO | 0.30 | 0.12 |  |  |  |  |  |  |  |  |
| 4. P-Education | 1 | 0 | 0.17 |  |  |  |  |  |  |  |
| 5. P-Avoidance | -0.06 | -0.10 | 0 | -0.01 |  |  |  |  |  |  |
| 6. P-AR | -0.10 | 0 | -0.30 | 0 | 0.15 |  |  |  |  |  |
| 7. C-Tenacity | 0 | 0.04 | 0.09 | 0.12 | -0.05 | -0.02 |  |  |  |  |
| 8. C-Strength | 0.06 | 0 | 0.02 | 0 | 0 | 0 | 0.53 |  |  |  |
| 9. C-Optimism | 0.08 | 0.06 | 0.02 | 0.09 | -0.16 | -0.21 | 0 | 0.22 |  |  |
| 10. C-FC | 0.50 | 0.14 | 0.10 | 0 | 0 | -0.06 | 0.11 | 0.15 | 0 |  |

Table S9 Family resilience and its influencing factors network edge weight in offspring caregivers (n=103)

|  | 1 | 2 | 3 | 4 | 5 | 6 | 7 | 8 | 9 | 10 |
| --- | --- | --- | --- | --- | --- | --- | --- | --- | --- | --- |
| 1. C-FCPS |  |  |  |  |  |  |  |  |  |  |
| 2. C-USR | 0.11 |  |  |  |  |  |  |  |  |  |
| 3. C-MPO | 0.55 | 0.11 |  |  |  |  |  |  |  |  |
| 4. P-Education | 0.15 | 0.18 | 0 |  |  |  |  |  |  |  |
| 5. P-Avoidance | -0.08 | 0 | -0.08 | -0.10 |  |  |  |  |  |  |
| 6. P-AR | -0.12 | 0 | -0.09 | -0.04 | 0.41 |  |  |  |  |  |
| 7. C-Tenacity | 0.07 | 0.10 | 0 | 0.01 | -0.12 | 0 |  |  |  |  |
| 8. C-Strength | 0.06 | -0.004 | 0.06 | 0 | 0 | -0.02 | 0.54 |  |  |  |
| 9. C-Optimism | 0.004 | 0.17 | 0.05 | 0 | 0 | -0.12 | 0.07 | 0.13 |  |  |
| 10. C-FC | 0.35 | 0.02 | 0 | 0 | 0 | -0.13 | 0.05 | 0.08 | 0.26 |  |

Figure S16 Comparison of centrality indices of family resilience and its influencing factors network among spouse caregivers and offspring caregivers(n=221)

a spouse caregivers (n =101)

b offspring caregivers (n =103)

Figure S17 Bootstrapped difference test node strength.

a spouse caregivers (n =101)

b offspring caregivers (n =103)

Figure S18 Bootstrapped difference test edges.

a spouse caregivers (n =101)

b offspring caregivers (n =103)

Figure S19 Bootstrapped confidence intervals of estimated edge-weights for the estimated network.

a spouse caregivers (n =101)

b offspring caregivers (n =103)

Figure S20 Stability of centrality indices by case dropping subset bootstrap.
